# Crossing Total Occlusions: Navigating Towards Recanalization

**DOI:** 10.1007/s13239-016-0255-0

**Published:** 2016-02-01

**Authors:** Aimée Sakes, Evelyn Regar, Jenny Dankelman, Paul Breedveld

**Affiliations:** Department of Biomechanical Engineering, Faculty of Mechanical, Maritime and Materials Engineering, Delft University of Technology, Mekelweg 2, 2628 CD Delft, The Netherlands; Department of Biomedical Engineering, Erasmus Medical Center, PO Box 2040, 3000 CA Rotterdam, The Netherlands

**Keywords:** Chronic total occlusions (CTO), Crossing, Percutaneous coronary intervention (PCI), State of the art, Treatment, Recanalization, Review

## Abstract

Chronic total occlusions (CTOs) represent the “last frontier” of percutaneous interventions. The main technical challenges lies in crossing the guidewire into the distal true lumen, which is primarily due to three problems: device buckling during initial puncture, inadequate visualization, and the inability to actively navigate through the CTO. To improve the success rate and to identify future research pathways, this study systematically reviews the state-of-the-art of all existing and invented devices for crossing occlusions. The literature search was executed in the databases of Scopus and Espacenet using medical and instrument-related keyword combinations. The search yielded over 840 patents and 69 articles. After scanning for relevancy, 45 patents and 16 articles were included. The identified crossing devices were subdivided based on the determinant for the crossing path through the occlusion, which is either the device (straight and angled crossing), the environment (least resistance, tissue selective, centerline, and subintimal crossing) or the user (directly steered and sensor enhanced crossing). It was found that each crossing path is characterized by specific advantages and disadvantages. For a future crossing device, a combination of crossing paths is suggested were the interventionist is able to exert high forces on the CTO (as seen in the device approach) and actively steer through the CTO (user: directly steered crossing) aided by intravascular imaging (user: sensor enhanced crossing) or an intrinsically safe device following the centerline or path of least resistance (environment: centerline crossing or least resistance crossing) to reach the distal true lumen.

## Introduction

It is often stated that chronic total occlusions (CTOs) represent the “last frontier” of percutaneous coronary interventions (PCIs).^55^ This statement is substantiated by the fact that PCIs in CTOs have considerably lower procedural success rates, in between 55 and 90%, than those achieved in non-occluded or acutely occluded coronary vessels (>95%), with the higher success rates in the hands of a few dedicated expert operators.^19^,^51^,^55^ The main contributor to the lower success rates seen in PCIs of CTOs is the technically challenging procedure, which requires a long learning curve and high technical skill from the interventional cardiologists. Even though recent advances in guidewire, catheter, and crossing device technologies have steadily increased the technical and procedural success rates of PCIs in CTOs over the last 5 years, CTOs still remain the lesion subtype in which PCI is most likely to fail.^55^ Therefore, improvement is still required to reach a widespread 95% success rate of PCIs in CTOs, for even the less experienced operators.

The main technical challenge during PCIs in CTOs, accounting for approximately 80% of procedural failure, lies in guidewire crossing into the true lumen of the distal vessel primarily due to three problems.^55^ Firstly, currently available equipment, including guidewires and crossing devices, are often unable to physically cross the tough fibrous cap of the CTO. The small diameter (<0.4 mm) and flexibility of the guidewire (and crossing devices alike) result in limited bending stiffness. Attempting to penetrate the tough proximal cap, therefore, often causes buckling. Since acute lesions are softer and have no fibrous cap, buckling is usually not observed, explaining the higher success rate. Secondly, even if the initial puncture is successful, crossing the CTO remains challenging due to inadequate 3D visualization during the crossing procedure. This inadequate visualization complicates navigating and can, therefore, cause blood vessel wall trauma, false lumen creation, and even discontinuation of the procedure due to uncertainty about the position of the guidewire.^51^,^55^ Thirdly, the inability to actively navigate across the CTO to, for example, compensate for guidewire deflection by heavily calcified regions or cross highly tortuous vessels, complicates reaching the distal true lumen.

Based on these three main challenges in PCIs of CTOs an international panel of 47 physicians has drafted three main requirements a CTO crossing device must meet to increase the success rate.^54^ First of all, the crossing device should be able to move forward even through resistant fibrotic and calcified tissue, either through mechanical means or by using an energy source. Secondly, the crossing device should be able to detect and ensure correct intraluminal passage. Finally, the crossing device should be able to precisely steer through the CTO.

Despite advances in equipment, with crossing devices incorporating at least one of the abovementioned functionalities, CTO recanalization may still be unsuccessful in approximately 25% of cases.^55^ As of today, a device incorporating all three of the proposed functionalities is still a work-in-progress. Therefore, it is a necessity to fundamentally explore new ways to safely and effectively cross CTOs and incorporate the three main functionalities that could in future increase the success rate in PCIs of CTOs.

A review of the state-of-the-art in crossing devices could potentially give insight into a future crossing device that incorporates all these functionalities. Even though some reviews exists that describe the current state-of-the-art in crossing devices specifically designed for CTOs, these reviews are incomplete as they mainly focus on providing an overview of currently applied devices; excluding the patented literature.^30^,^55^,^63^ This study explores the entire field, including the patented literature, and systematically reviews the state-of-the-art of all existing and invented crossing devices and methods for crossing total occlusions, including acute occlusions (which are usually softer (thrombotic) than CTOs, which are mainly characterized by heavy calcification), used in clinical practice and designs described in the patented literature. The study ends in a discussion in which we identify future research pathways that could lead to a fundamental improvement in the field.

## Literature Search Method

A literature search was executed in the database of Scopus and Espacenet and was limited to the English or Dutch language from the 1950s to the present. The search was broadened to include all kinds of occlusions, including acute and semi-occluded lesions, to get a complete overview of all the devices available for crossing obstructions in the vascular system.

The search terms in the Scopus search engine were subdivided into four categories: (1) occlusion, (2) treatment, (3) medical area, and (4) instrument type. In the occlusion category, the search terms: occlu*, obstruct*, plaqu*, thromb*, *clot*, obstacle*, and barrier* were used. The treatment category included: *canal*, remov*, resect*, dissect*, and cut*. In the area category, the following terms were used: vasc*, cardio*, arter*, vessel*, vein*, and cappilar*, and in the instrumental category: device*, instrument*, prototype*, guidewire*, catheter*, and apparatus*. The categories were connected with the “AND” operator; the search terms either with “AND” or “OR”. The “NOT” operator was added to filter out non-relevant articles.

The final literature search in Scopus led to 69 hits. Of these hits, first the titles were scanned for relevancy, after which the abstract was read. If it was concluded that the article fitted the scope of this review as discussed in the previous section, it was included in this review. This led to a total of 16 articles being included in this review.

Subsequently, the Espacenet database was searched for patents relating to crossing devices for occlusions using the following keywords in the title and in the title and abstract, respectively: (occlu* OR obstruct* OR plaqu* OR thromb* OR clot*), and ((vasc* OR vessel*) AND (canal* OR remov* OR resect*)). This led to 845 potentially relevant patens. By first reading the title of the patents, followed by the summary of the invention, a total of 42 were selected.

## State of the Art Devices

**Figure 1:**
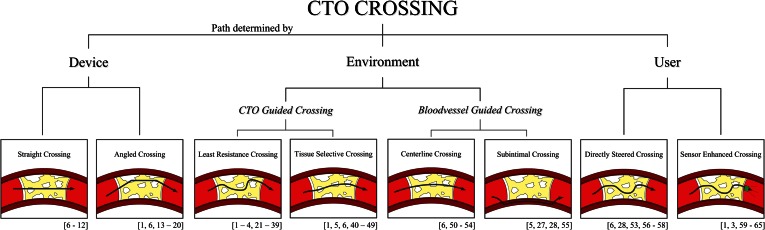
CTO crossing methods—overview. Per crossing method the associated references are illustrated in between brackets. Color indications: Red = blood vessel. Yellow = plaque material. White = calcified regions. Green = sensor.

It was found that there are multiple ways or methods to cross an occlusion and reach the true lumen at the distal (or proximal) end of the blood vessel. Which path is followed depends on the chosen crossing device. Three main approaches can be distinguished: the *Device*, *Environment*, and *User* approach (see Fig. 1). Per approach, the associated devices will be discussed and the intended use, i.e., for acute occlusions or CTOs, and current status, i.e., abandoned, in use, or proposed (amongst others) will be indicated.

### Device

In the *Device* approach, the crossing device itself is the most determining factor for the crossing path. The occlusion is crossed in a straight or angled manner, independent of the properties and geometry of the occlusion and blood vessel, as well as the input from the user, called *Straight Crossing* or *Angled Crossing*, respectively.

#### Straight Crossing

**Figure 2:**
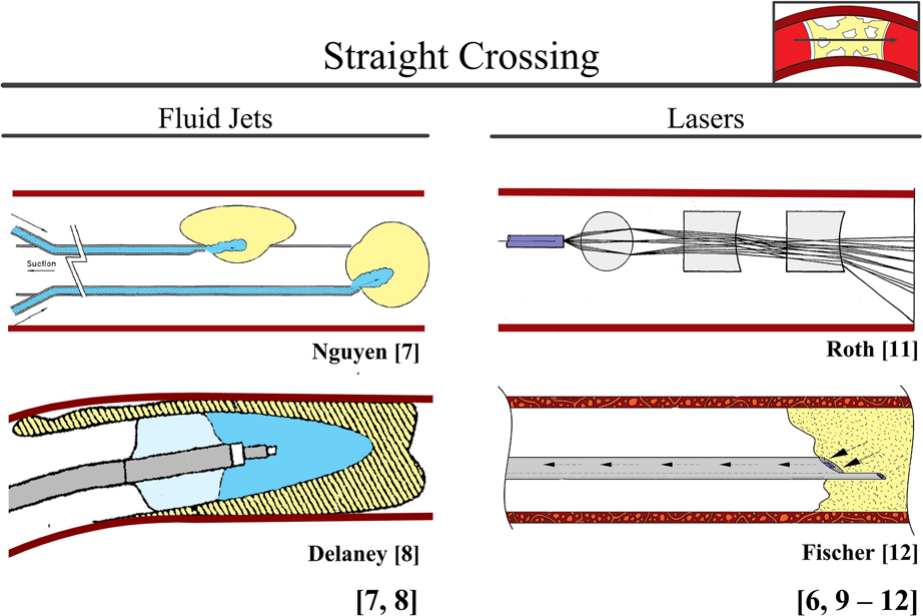
Device: straight crossing. Color indications: Red = blood vessel wall. Yellow = plaque material. Light blue = balloon. Blue = liquid. Purple = laser.

Developed devices that cross the CTO in a straight line use a fluidic (fluid jets) or gaseous medium (lasers) (Fig. 2). The initial orientation of the tip of these devices determines the crossing direction. It is, therefore, imperative that the tip of the device is perpendicular to the CTO cap and, as best as can be achieved, collinear to the lumen. The crossing speed is controlled by the input power of the laser or fluid jet.

##### Fluid Jets (Abandoned, Originally in Use for Acute Occlusions)

In hydrodynamic thrombectomy, a high-velocity fluid jet is used to fragment and remove occlusive material from the blood vessel wall. For this purpose, high-velocity fluid jets use a fine stream of saline at high pressures (typically between 15 and 20 kg/cm^2^). Currently, fluid jets used for crossing purposes are abandoned due to safety concerns (note that some still exist for treating purposes). However, Nguyen *et al.*^22^ and Delaney *et al.*^11^ propose axially directed fluid jets for crossing acute occlusions (Fig. 2).

##### Lasers (in Use for Peripheral and Coronary CTOs)

Medical lasers use a high-energy beam of light to resect or dissolve different tissue types for crossing or debulking purposes. Absorption of the laser energy within the targeted biological tissues creates photochemical and photomechanical reactions that result in conversion of the plaque material in vapor and the development of acoustic shock waves that are able to fragment tissue.

Lasers, such as over-the wire and rapid-exchange excimer laser catheters, are currently in use for CTO crossing in atherectomy. These laser catheters contain a flexible fiber-optic cable made out of as many as 240 high-purity silica fibers arranged around a guidewire lumen, with the distal tip polished and rounded. Examples of laser crossing systems are the *CVX*-*300 Excimer Laser* (Spectrametrics Inc., Colorado Springs, CO) and the *TURBO elite laser ablation catheter* (FDA approved October, 2006).^63^ Furthermore, in^3^,^18^,^40^,^44^ different lasers are proposed for crossing acute occlusions and CTOs (see Fig. 2 for the laser devices proposed by Roth *et al.*^44^ and Fischer *et al.*^18^).

Unfortunately, heat accumulation is often an issue in lasers, warranting careful advancement through the CTO, slower than 1 mm/s, to increase absorption of the plaque and prevent absorption by the blood vessel wall.^63^ To minimize this problem, Pallarito *et al.*^40^ and Roth *et al.*^44^ suggest using focusing devices (lenses) to narrow the laser beam (see Fig. 2 for the device proposed by Roth *et al.*). Despite the drawback of heat accumulation, a major advantage of lasers is that buckling is no issue as there are no mechanical forces on the device tip.

#### Angled Crossing

Many of the current and proposed crossing devices have a rigid tip (*L* > 2 mm). Since rigid tips do not allow for bending or compression, the tips of these devices cannot adjust to the 3D shape and direction of the CTO. In the flexible device shaft behind the rigid tip, however, bending is possible, which allows for some adjustment to bends in the vascular system when high radial resistance is encountered. The route through the CTO is, therefore, not smooth, but contains multiple acute angles, which from here on out will be referred to as *Angled Crossing*.

##### Hinged Crossing Device (in Use for Peripheral and Coronary CTOs)

**Figure 3:**
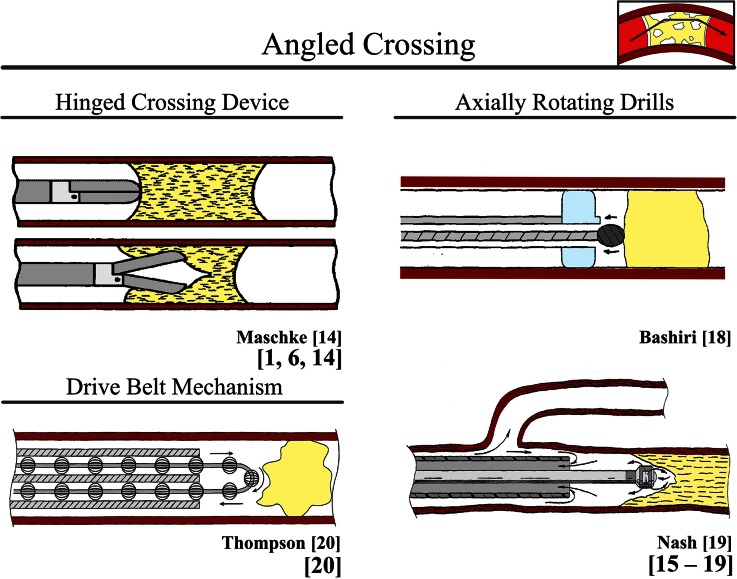
Device: angled crossing. Color indications: Red = blood vessel wall. Yellow = plaque material. Light Blue = balloon.

*Frontrunner XP* (Ø0.76–1 mm, 2.8 F distal tip, Cordis Corporation, Miami, FL)^55^ is an FDA-approved crossing device specifically designed for crossing heavily calcified CTOs.^9^*Frontrunner XP* uses a hinged bilateral distal tip assembly to cross and subsequently treat the occlusion *via* the principle of blunt microdissection (see Fig. 3 for a similar device is described by Maschke *et al.*^33^).^33^,^55^ According to Mossop *et al.*,^63^ procedural success of up to 91% can be achieved in peripheral CTOs.

##### Axially Rotating Drills (in Use for Peripheral CTOs and Acute Occlusions)

In atherectomy, axially rotating drills are used to resect and remove plaque from the blood vessel wall. There are three main types of axially rotating drill atherectomy devices: orbital, rotational, and directional. The three main drill types mainly differ in drill bit design.

Current FDA-approved rotating drill atherectomy devices are the *Clot Buster Amplatz Thrombectomy Device catheter* (Ø2.7 mm, *L* = 50 and 120 cm; ATD, Microvena, White Bear Lake, MN; in use for peripheral acute occlusions) (Fig. 3)^12^ and the *Wildcat catheter* (Avinger, Redwood City, CA; in use for peripheral CTOs).^42^ Success rates of 83 and 89% are reported in peripheral total occlusions with *ATD* and *Wildcat catheter*, respectively.^42^*ATD* is actuated by compressed air, rotating a shielded helical cutter at 150,000 rpm.^12^ This rotation causes negative pressure in close proximity to the cutter, which sucks the occlusive material towards the drill to macerate it. The *Wildcat catheter*, in comparison, is rotated by hand and driven by a flexible drive shaft. The tip can be altered while inside the body from a passive into an active (more aggressive) configuration. Furthermore, in^5^,^23^,^37^ multiple additional rotating drill atherectomy devices are proposed for crossing acute (thrombotic) occlusions and CTOs (see Fig. 3 for the drill designs proposed by Bashiri *et al.*^5^ and Nash *et al.*^37^).

##### Drive Belt Mechanism (Proposed for Acute Occlusions)

Thompson *et al.*^57^ propose a drive belt mechanism equipped with abrasive devices that shave off and remove small amounts of clot material at a time (Fig. 3).

### Environment

In the *Environment* approach, the direct environment around the device is the determinant for the crossing path. The environment around the device consists of the CTO and the blood vessel (wall). In *CTO Guided Crossing*, the difference between biomechanical properties throughout the CTO is the determining factor for the crossing path. In *Blood VesselGuided Crossing*, the blood vessel is used as a guide for crossing.

#### CTO Guided Crossing

Two different CTO guided crossing paths can be distinguished: *Least Resistance Crossing* and *Tissue Selective Crossing*. In the first approach, less (pressure) resistant tissue types, such as fat, or micro vessels are used to cross the occlusion. This method is, amongst others, used by guidewires; the most common crossing device used in PCI. In the second approach, specific tissue types are targeted, such as calcium, fibrin, or fat, to cross the CTO. Which tissue is targeted depends on the type of crossing device used.

##### Least Resistance Crossing

###### Guidewires (in Use for Coronary CTOs, Peripheral CTOs, and Acute Occlusions)

Guidewires are thin (Ø0.22–0.40 mm for coronary application^54^) flexible wires that are inserted into the vascular system to cross occlusions (CTOs and acute occlusions) and guide other endovascular instruments, such as support and balloon catheters, typically during angioplasty. They usually consist of an inner core and outer spring-coil or polymer jacket.^51^

As of today, guidewires remain the crossing tool of choice during PCI. Many of the discussed devices can be used in conjunction with a guidewire, but this is not a prerequisite. Over the last 5 years, the introduction of better guidewire designs (with higher tip loads, i.e., load in grams [g] at which the guidewire start to buckle, amongst others) has drastically improved the success rate of PCIs in CTOs.^51^,^55^ Dedicated CTO guidewire designs differ based on core design, the presence or absence of a polymer cover, tip design, and the type of coating (Table 1).^51^

**Table 1:** Dedicated guidewires frequently used in the treatment of CTOs

Manufacturer	CTO guidewire	Diameter (inch/mm)	Core material	Tapering (inch/mm)	Tip design	Polymer cover	Coating	Tip load (g)
Abbott Vascular	Cross-it™ 100, 200, 300, and 400	0.014/0.35	SS	Y [0.001/0.25]	C2T	N	HI	2, 3, 4, and 6
	Whisper™ LS, MS, and ES	0.014/0.35	(Durasteel) SS	N	C2T	Y	HI	1
	Pilot™ 50, 150, 200	0.014/0.35	(Durasteel) SS	N	C2T	Y	HI	2, 4, and 6
	Progress™ 40, 80, 120, 140T, and 200T	0.014/0.35	(Durasteel) SS	140T [0.010/0.25] 200T [0.009/0.23]	C2T	I	HI	4.8, 9.7, 13.9, 12.5, and 13.9
Asahi Intecc Corporation	Intermediate™	0.014/0.35	Data unavailable	N	C2T	N	HO	3
	Miraclebros™	0.014/0.35	Data unavailable	N	C2T	N	HO	3, 4.5, 6, 9, and 12
	Confianza™, Confianza™ Pro, and Confianza™ Pro “8–20”	0.014/0.35	Data unavailable	[0.009/0.23] Pro “8–20” [0.008/0.20]	C2T	N	HY	9, 12, and 20
	Fielder™, Fielder™ FC, and Fielder™ X-Treme	0.014/0.35 X-Treme 0.014/0.33	SS	X-Treme [0.009/0.23]	C2T	Y	HI	1, 0.8, and 0.8
	Gaia™ 1st, 2nd, and 3rd	0.014/0.36	SS	1st [0.010/0.26] 2nd [0.010/0.28] 3rd [0.012/0.30]	C2T	N	HI	1.7, 3.5, and 4.5
Boston Scientific	Choice™ PT and PT2 LS and MS	0.014/0.35	SS	N	C2T	Y	HI	2
	PT Graphix™ Intermediate Graphix P2™ LS and MS	0.014/0.35	SS	N	SR	Y	HI	3 and 4
Cordis/Johnson & Johnson	Shinobi™ and Shinobi™ Plus	0.014/0.35	Data unavailable	N	C2T	N	HO	2 and 4
Medtronic	Persuader™ 3, 6, and 9	0.014/0.35	Data unavailable	9 [0.011/0.28]	C2T	N	HI	3, 6, and 9
Terumo	Crosswire™ NT, Hard type 40, and Hard type 80	0.014/0.35	Nitinol	N	C2T	N	HI	5.5, 15.6, and 26.7
	Runthrough™ NS Floppy, NS Hypercoat*, and NS intermediate	0.014/0.35	Nitinol	N	C2T	N(Y*)	HI	1, 1, and 3.6
	Glidewire™ Gold Neuro	0.011/0.27	Nitinol	N	C2T	N	HI	1

Tip load is defined as the load in grams (g) at which the guidewire starts to buckle. Table partly adapted from^51^

Durasteel, high tensile stainless steel; SS, Stainless Steel; N, no; Y, yes; C2T, Core 2 tip; SR, Shaping ribbon; I, Intermediate; HI, Hydrophilic; HO, hydrophobic; HY, hybrid, hydrophilic shaft, uncoated tip

Cores of contemporary dedicated CTO guidewires differ based on the diameter (with higher tip loads with increasing core diameter), the material used; stainless steel or nitinol, and the presence of core tapering. CTO guidewires with stainless steel cores, such as *Whisper* (Abbott Vascular, Abbott Park, IL, USA) and *Pilot* ((Abbott Vascular, Abbott Park, IL, USA), provide excellent support and tracking abilities (i.e., the ability of the guidewire body to follow the tip around bends). However, they are less flexible and more susceptible to buckling (i.e., have lower tip loads) than guidewires with nitinol cores, such as *Crosswire* (Terumo, Tokyo, Japan).^4^,^15^

**Figure 4:**
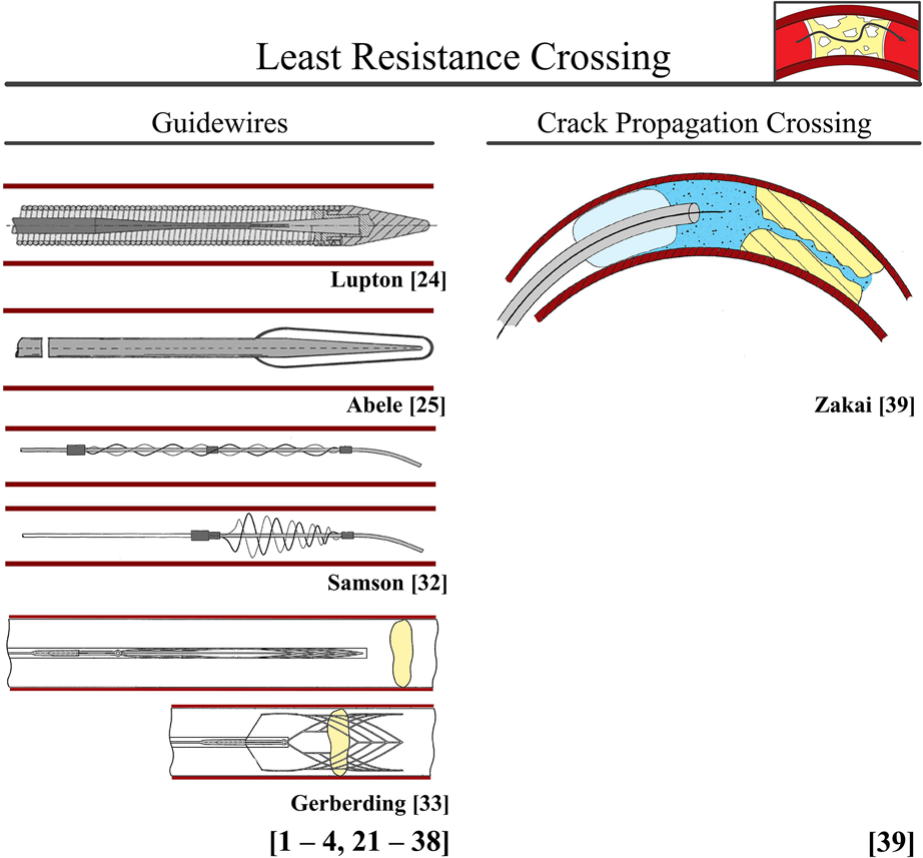
Environment CTO: least resistance crossing. Color indications: Red = blood vessel wall. Yellow = plaque material. Light blue = balloon. Blue = liquid.

Another important factor influencing the tip load, support, and trackability of a guidewire is core tapering. Gradual or long tapers (in, for example, *Gaia* (Asahi Intecc. Corp., Nagoya, Japan)) provide less support and lower tip loads, but show greater trackability than short tapers (in, for example, *Confianza* (Asahi Intecc. Corp., Nagoya, Japan)). Tapered core designs are also proposed by^31^,^39^ (see Fig. 4 for the design proposed by Lupton *et al*.^31^).

Placed over the core is either an outer coil or polymer cover.^15^ An outer coil (such as in *Persuader* (Medtronic, Minneapolis, MN, USA)) adds flexibility to the distal part of the guidewire and affects the support, tracking, and visibility. Instead of outer coils, some guidewires have a polymer or plastic covering over the tapered wire core (such as in *Whisper* (Asahi Intecc. Corp., Nagoya, Japan)). Polymer covers provide smooth tracking through torturous blood vessels.

Two different tip designs can be distinguished:^15^ the core to tip (C2T) design (in which the core of the guidewire is fully extended to the tip (such as in *Runthrough NS* (Terumo Medical Corp., Tokyo, Japan))) and the shaping ribbon (SR) design (in which the core is not fully extended to the tip; instead a small piece of metal bridges the gap between the end of the core and the tip (such as in *PT Graphix Intermediate* (Boston Scientific, Natick, MA, USA))). The C2T design results in a more responsive guidewire, whereas the SR design is characterized by a more atraumatic flexible tip. Additionally, enlarged resilient tip portions (including the Magnum wire (Schneider, Zurich, Switzerland))^1^,^34^ (see Fig. 4 for the design proposed by Abele *et al.*^1^) are proposed to increase the tip load.

In order to decrease the sliding friction, low friction coefficient guidewires containing a hydrophilic (HI), hydrophobic (HO; polymeric), or hybrid (HY) coating (e.g., *Confianza* (Asahi Intecc. Corp., Nagoya, Japan)) are currently available. Hydrophilic coatings attract water and become gelatinous when wet, reducing friction, while hydrophobic coatings repel water, and also reduce friction, but not to the same extent as hydrophilic wires.^51^ Available dedicated CTO guidewire types with a hydrophilic polymeric coating on the tip are (amongst others) *Fielder* (Asahi Intecc, Nagoya, Japan) and *ConquestConfianza* (Asahi Intecc, Nagoya, Japan).^7^,^19^,^20^ Additionally, two patents^1^,^24^ discuss the use of a hydrophilic coating, and Fearnot *et al.*^17^ suggest a guidewire with an antithrombotic coating to decrease friction by dissolving the occlusion in direct contact with the guidewire.

According to Sianos *et al.*,^51^ the dedicated CTO guidewires should be used in conjunction with a micro catheter in order to prevent flexion and buckling (by improving its columnar strength), and as such improve the chance of a successful crossing procedure. Currently, several different (micro) guiding catheters, such as *Tornus* (Asahi Intecc, Nagoya, Japan), *CrossBoss* (Boston Scientific, Natick, MA, USA), and *Corsair* (Asahi Intecc, Nagoya, Japan) are available that have proven successful in increasing the buckling resistance of dedicated guidewires. Furthermore, Montague *et al.*^36^ also proposes to use an introducer sheath (or micro catheter) to improve the columnar strength of the guidewire.

Another challenge in PCI of CTOs is the balloon uncrossable occlusion, in which the guidewire successfully crosses the occlusion, but neither a ballooncatheter nor microcatheter can be advanced through the CTO, seen in about 2% of the failure cases.^51^,^55^ If this is encountered, several strategies can be applied:^51^ (1) a second stiff guidewire can be placed proximal to the CTO (preferably in a side-branch), (2) a second balloon can be inflated proximal to the CTO (preferably in a side branch), or (3) the balloon or microcatheter can be exchanged for a *Tornus* (Asahi Intecc, Nagoya, Japan) to enlarge the lumen. Furthermore, to overcome this problem altogether, multiple patents describe combined crossing and treatment tools (see Fig. 4 for the devices proposed by Samson *et al.*^47^ and Gerberding *et al.*^21^).^14^,^21^,^24^,^29^,^46^–^48^,^56^ In these designs, a cable-actuated or self-expandable cage-like structure is described that in collapsed state functions as a guidewire and in expanded state as a treatment device, similar to a stent.

###### Crack Propagation Crossing (Proposed for Peripheral CTOs, Coronary CTOs, and Acute Occlusions)

In the crack propagation crossing method, proposed by Zakai *et al.*^64^ (see Fig. 4), an elevated pressure between the crossing device and occlusion is used to “crack” or fragment the occlusion at its weakest region. Crack formation and propagation along the occlusion follows the path of least resistance until the distal side is reached and the pressure drops.

##### Tissue Selective Crossing

###### Spark Erosion (Proposed for Coronary CTOs)

**Figure 5:**
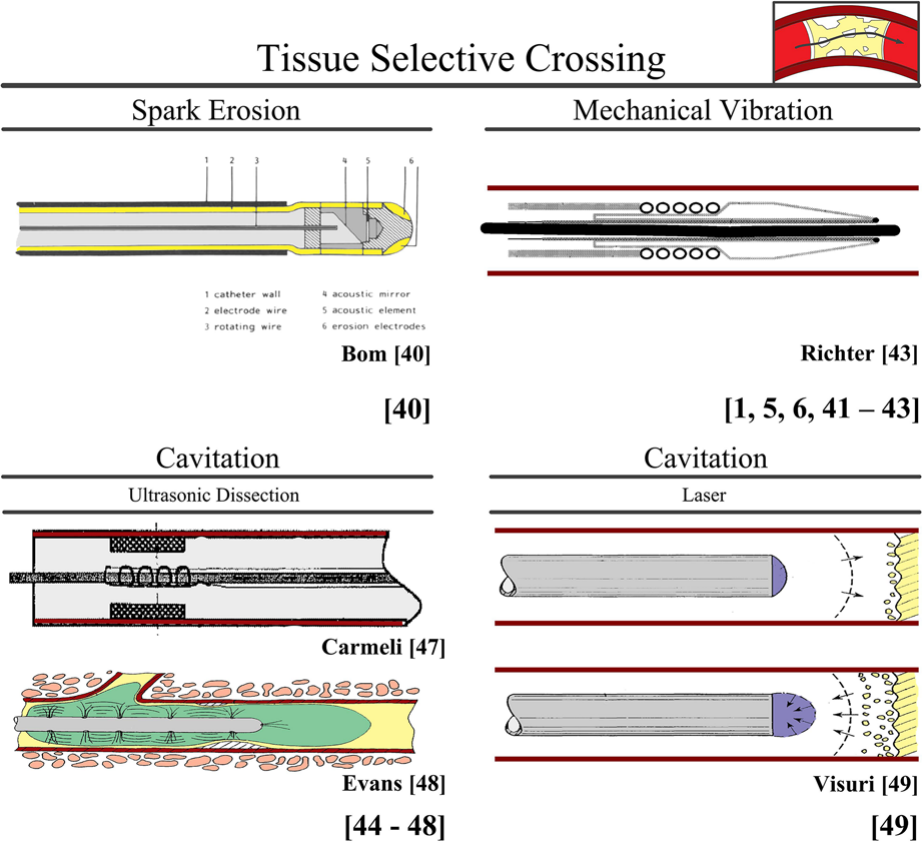
Environment CTO: tissue selective crossing. Color indications: Red = blood vessel wall. Yellow = plaque material. Bright Yellow (in upper left schematic illustration) = electrodes. Purple = laser. Green = antithrombotic agent.

In the spark erosion crossing method proposed by Bom *et al.*,^6^ two electrodes, placed in close proximity to the occlusion, generate sparks to fragment and cross occlusions (Fig. 5). In this method, the electric conductivity of the tissue types determines the crossing path. As less electrically conductive materials, such as calcium, are not fragmented, whereas those that are electrically conductive, such as collagen and blood, are fragmented, this crossing method is less suitable for highly calcified CTOs.

###### Mechanical Vibration (in Use for Peripheral and Coronary CTOs)

In the mechanical vibration crossing method it is hypothesized that selective penetration depends on the difference in elasticity between the different tissue types. Collagen rich structures, such as the blood vessel wall, are not damaged by vibrational energy as they are elastic and, therefore, move out of the way. In contrast, less elastic atherosclerotic plaque tissue is not able to move out of the way and is thus fragmented.

Mechanical vibration energy is used in vibrational angioplasty. A current FDA-approved vibrational angioplasty device for crossing CTOs is the *Crosser Catheter* (originally developed by FlowCardia, Sunnyvale, CA, currently by BARD Peripheral vascular Inc., Tempe, AZ, USA) that consists of a nitinol transmission wire with a blunt tip and a generator.^25^,^30^,^65^ The *Crosser Catheter* uses high frequency (21 kHz) low-amplitude vibration energy to break through the cap and subsequently the CTO. A success rate of in between 40 and 75% was reported with this device in peripheral CTOs.^25^,^30^ In another currently available vibrational angioplasty device designed by Medical Miracles (UK), a success rate of in between 75 and 77.4% was reported in coronary CTOs.^55^,^63^ In this device, a conventional coronary angioplasty guidewire is guided through a catheter and vibrated using reciprocal and lateral movements with frequencies between 16 and 100 Hz.^55^,^63^ Richter *et al.*^43^ propose a similar device for crossing, in which a distal attachment mechanism couples a guidewire to the catheter and allows for a vibrating-generating means to add axial vibrating motion to the guidewire tip (see Fig. 5).

###### Cavitation (Proposed for Peripheral CTOs, Coronary CTOs, and Acute Occlusions)

In the cavitation crossing method, small cavitation bubbles are used to fragment specific tissue types. These cavitation bubbles are formed by rapid blood pressure changes, which cause the bubbles to implode, creating shockwaves that are able to fragment tissue. Tissues with high water content (such as fat) or brittle tissues (such as calcium) are fragmented, but vessels and nerves, which have high collagen content, are preserved.

*Cavitation—Ultrasonic Dissection* In ultrasonic dissection, cavitation bubbles are created using longitudinal vibrations generated by an ultrasonic generator (e.g., a piezoelectric crystal or a magnetic field). Siegel *et al.* describes a series of experiments in peripheral CTOs with an ultrasonic probe system consisting of a piezoelectric transducer, multiple titanium wires to transfer the ultrasonic energy to the tip of the device, and a Ø2.0 mm ball-shaped tip.^52^ Similar devices are proposed by Wang *et al.*^61^ (for acute occlusions), Nita *et al.*^38^ (for all occlusion types), and Carmeli *et al.* (for CTOs)^8^ (see Fig. 5 for the design proposed by Carmeli *et al.*^8^). Siegel *et al.*^52^ showed that heavily calcified regions resist recanalization with the ultrasonic probe, making this method less suited for crossing older, more calcified, CTOs. To increase the effectiveness of ultrasonic dissection in acute occlusions, Evans *et al.*^16^ propose the addition of thrombolysis (see Fig. 5).

*Cavitation—Laser* Another method to create cavitation bubbles for tissue fragmentation is a laser, as proposed by Visuri *et al.*^60^ (see Fig. 5). In this technique, small-pulsed burst of laser light are used to create cavitation bubbles that are able to fragment tissues with high water content.

#### Blood Vessel Guided Crossing

In *Blood Vessel Guided Crossing*, the blood vessel wall is used for support during the crossing procedure. A subdivision is made between crossing devices that use the blood vessel wall to cross *via* the centerline of the blood vessel, called *Centerline Crossing*, and crossing devices that cross subintimally, i.e., between the intima and adventitia of the blood vessel wall, and use the support of these layers of the blood vessel wall for crossing, called *Subintimal Crossing*.

##### Centerline Crossing

Centerline following can be achieved with expanding or elastic self-centering mechanisms. In these devices the blood vessel wall is used for supporting and centering the crossing device. Not only does this support prevent blood vessel wall dissection or puncture, it also increases the buckling resistance of the tip of the guidewire or crossing device by increasing the effective diameter of the device. It must be noted, however, that if the crossing device is not translated exactly forward with the self-centering mechanism, true centerline following will be difficult in tortuous and longer occlusions.

###### Balloons (Undergoing Clinical Trials and Proposed for Peripheral CTOs)

**Figure 6:**
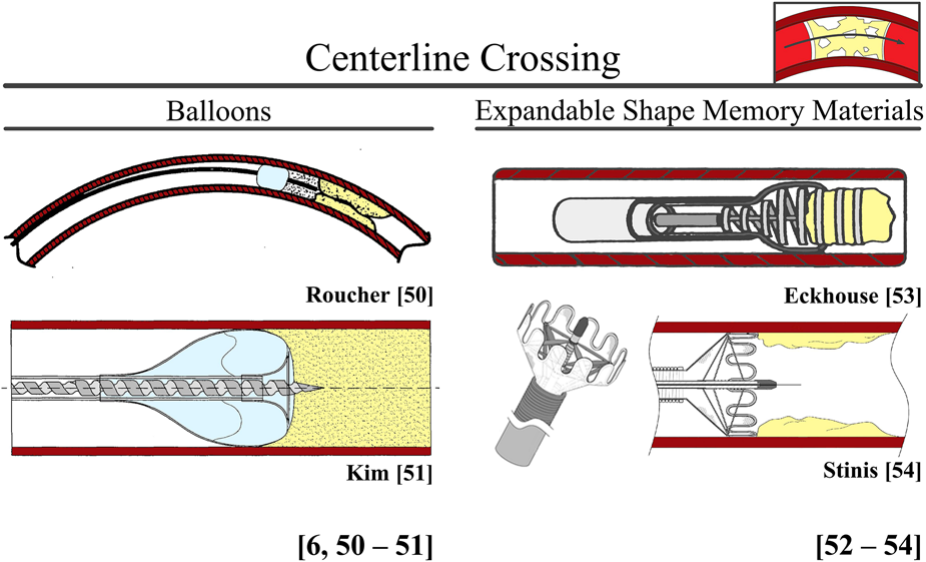
Environment blood vessel: centerline crossing. Color indications: Red = blood vessel wall. Yellow = plaque material. Light blue = balloon.

The centerline crossing *ENABLER*-*P Balloon Catheter System* (EndoCross Ltd., Yokneam, Israel), described by Buchbinder, is currently undergoing clinical trials for crossing peripheral CTOs.^63^*ENABLER*-*P* uses a specially designed support balloon that allows the balloon to elongate upon additional cyclical inflation, and as such moves a standard guidewire 3 mm forward without exceeding its inherent diameter. Multiple inflation/deflation cycles advances the guidewire forward through the occlusion. With this device, successful guidewire crossing was achieved in 86.4% of the cases.^63^ Furthermore, Roucher *et al.*^45^ and Kim *et al.*^26^ propose to use a balloon in combination with a central crossing tool for centerline crossing of CTOs (see Fig. 6).

###### Expanding Shape Memory Materials (SMA) (Proposed for Acute Occlusions)

Currently no clinically tool is available or undergoing clinical trails using expanding SMMs, such as shape memory alloys (SMAs) and shape memory polymers (SMPs). However, Vardi *et al.*,^59^ Eckhouse *et al*.,^13^ and Stinis *et al.*^53^ propose a mechanism using (self-) expanding SMAs, such as nitinol, to mimic the shape of the blood vessel wall in combination with a central crossing device (see Fig. 6 for the designs proposed by Eckhouse *et al.*^13^ and Stinis *et al.*^53^). Furthermore, in the designs of Eckhouse *et al.*^13^ and Stinis *et al.*^53^ the self-centering mechanism is translated forward with the crossing device, in principle allowing for true centerline following (Fig. 6).

##### Subintimal Crossing

Due to the stiff proximal and distal cap of the CTO, the guidewire or crossing device will sometimes penetrate the intima and cross the CTO subintimally (between the intima and adventitia of the blood vessel wall). During subintimal crossing, the support of both the intima and adventitia keeps the crossing device collinear to the direction of the blood vessel wall and prevents perforation. This support, however, makes reentry into the true lumen difficult and is often time consuming.^30^

Multiple specialized subintimal crossing and reentry devices are currently in use and being developed to improve reentry to the true lumen, including the *Outback Catheter* (Cordis Corporation, Bridgewater, NJ), the *Pioneer Catheter* (Medtronic Inc., Santa Rosa, CA), and the *Stingray Re*-*entry Device* (Boston Scientific, Natick, MA).^2^,^7^,^20^,^30^ These devices use a curved needle to puncture through the intima for reentry into the distal true lumen. This significantly increases the chance of successful reentry and thus the success rate of this crossing method.^30^

### User

In the *User* approach, all the control of the crossing path is given to the user. In this approach, the user of the crossing device is able to actively steer through the CTO, called *Directly Steered Crossing*. If the user so desires, heavily calcified regions can be circumnavigated. Furthermore, the addition of a tip sensor in *Sensor Enhanced Crossing* can give additional visual information about the position of the crossing device in relation to the direct environment. This information can, subsequently, be used as an extra navigational aid.

#### Directly Steered Crossing

##### Cable Actuation (in Use for Peripheral and Coronary CTOs)

Currently, a number of cable-actuated guidewires (≤0.4 mm) are available that can be actively steered through CTOs. The latest Cordis guidewires *Shinobi* and *Wizdom* enable tip deflection in one or two directions with a minimum bending angle of 45 degrees.^63^ Furthermore, the *Venture Catheter* (St. Jude Medical, MN) is a support catheter able to deflect all commercially available 0.36 mm guidewires with angles up to 90°.^63^

##### Electroactive Polymer Actuation (Proposed for Acute Occlusions)

**Figure 7:**
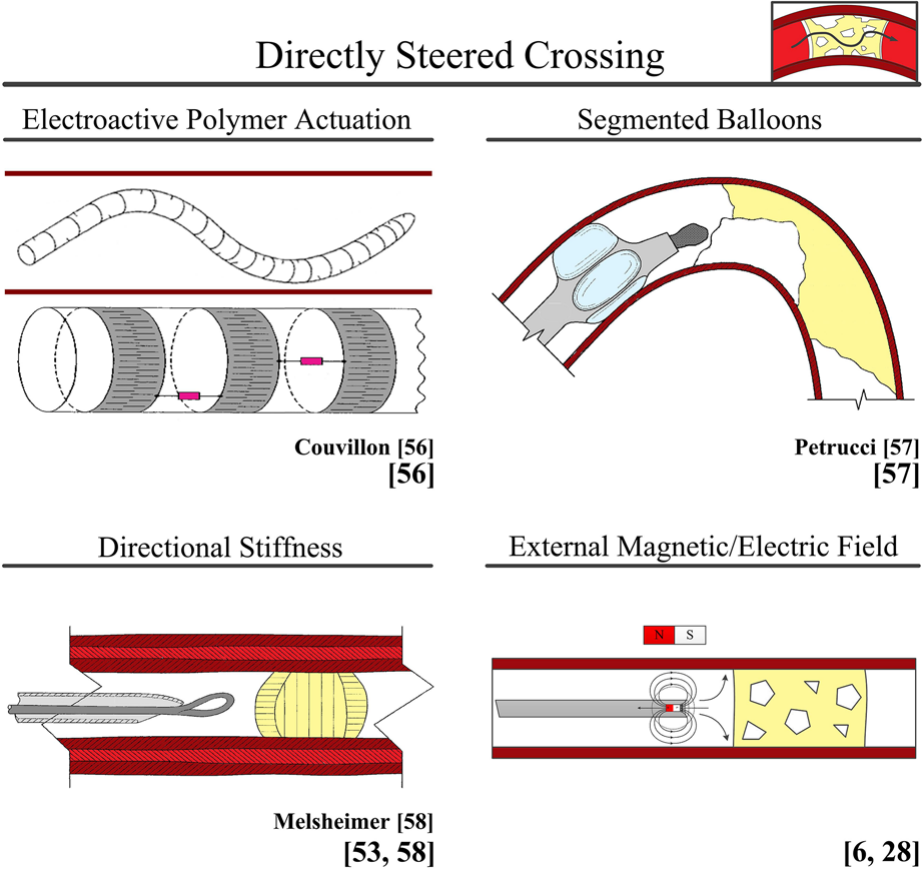
User: directly steered crossing. Color indications: Red = blood vessel wall. Yellow = plaque material. Light blue = balloon. Bright Pink = electroactive actuators.

Couvillon^10^ describes a directly steerable crossing device that uses multiple electroactive polymer actuators to steer the tip of the crossing device (see Fig. 7). These electroactive polymers act as joints, which enable direct steerability of the crossing device. By increasing the number of electroactive polymers, complex shapes such as three-dimensional s-curves can be achieved.

##### Segmented Balloons (Proposed for Peripheral CTOs, Coronary CTOs, and Acute Occlusions)

The device proposed by Petrucci^41^ uses a segmented intravascular balloon to steer the crossing device in plane (see Fig. 7). In plane steering can assure a central position with respect to the CTO, but does not allow for adjusting the tip orientation. A major advantage of this steering technique is, however, that by keeping contact with the blood vessel wall, the buckling resistance is increased.

##### Directional Stiffness (Proposed for Peripheral and Coronary CTOs)

Melsheimer *et al.*^35^ propose a guidewire with a specialized tip geometry for steering (see Fig. 7). In this approach, the difference in friction (between the tip and occlusion) caused by the orientation of the guidewire in the occlusion is used for steering. By rotating the guidewire, the tip orientation is changed and in this way the direction of motion. A similar approach is suggested by Eckhouse *et al*.^13^

##### External Magnetic/Electric Field (in Use for Peripheral and Coronary CTOs)

Magnetically or electrically enabled crossing tools have coils, magnets, or ferromagnetic materials incorporated inside their tip to enable tip deflection by an external electric or magnetic field outside the patient’s body (see Fig. 7 for a schematic representation of such systems). A currently available fully integrated magnetic navigation system for guidewires and catheters is the *Niobe MNS* (Stereotaxis, St. Louis, MO) with the associated FDA-approved magnetic *PowerAssert Radiofrequency Guidewire*.^63^ Examples of other magnetically enabled guidewires are *Titan* (Stereotaxis, St. Louis, MO) and *Pegasus* (Stereotaxis, St. Louis, MO).^7^

#### Sensor Enhanced Crossing

The determination of the precise 3D position and orientation of the CTO and crossing device is often difficult to determine with conventional *Computed Tomography* (*CT*) images. This uncertainty about 3D tip position of the crossing device can lead to blood vessel wall trauma, false lumen creation, and even discontinuation of the procedure. Therefore, there is a need for (intravascular) imaging methods that can give the user additional information about the orientation, position, and tissue types in front of the crossing device.

The latest advance in external cardiac imaging is *Multislice Computed Tomography* (*MSCT*). The main fundamental advantage of MSCT in comparison to conventional *CT* is the ability to visualize the CTO in 3D. Although *MSCT* is currently mainly used as a pre-operative imaging technique, the new generation 128-slice *MSCT* scanners are finding their way into the intervention room for real-time 3D imaging of the coronaries.^58^ Unfortunately, the resolution level is still relatively low and does not allow for reconstruction of thin intraluminal channels or thin collaterals.^51^ Furthermore, one of the major concerns in using *MSCT* is the radiation dose received by the patient.^51^

**Figure 8:**
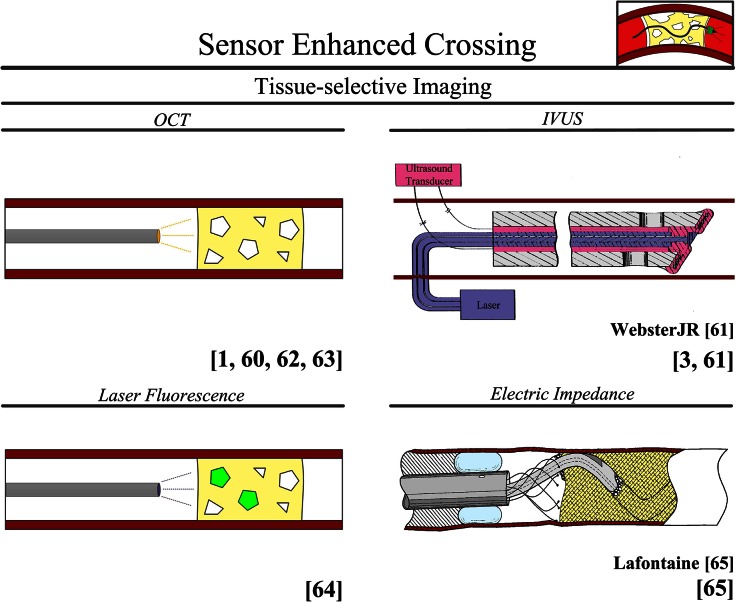
User: sensor enhanced crossing. Color indications: Red = blood vessel wall. Yellow = plaque material. Pink = ultrasound transducer. Light blue = balloon. Orange = optical coherence tomography imaging. Purple = laser. Bright Green = fluorescence. OCT, optical coherence tomography; IVUS, intravascular ultrasound.

The latest advances in non-iodizing intravascular cardiac imaging techniques (with a higher resolution than *MSCT*) are *Optical Coherence Tomography* (*OCT*) and *IntraVascular UltraSound* (*IVUS*) (see Fig. 8 for a schematic representation of both systems). In *OCT*, different tissue types are identified based on unique patterns of absorption, reflection, and scatter of near-infrared light.^27^ In *IVUS*, the difference in reflectance of ultrasonic sound waves is used to distinguish different tissue types.^62^

*OCT* (or Optical Coherence Reflectometry (*OCR*)) is currently applied as a tissue-selective (imaging) system in two FDA-approved micro-catheters: the *Safe*-*Cross Radiofrequency Total Occlusion Crossing* (*TOC*) *Guidewire System* (Ø0.36 mm, Intraluminal Therapeutics, Carlsbad, CA; in use for coronary CTOs)^27^,^50^,^55^ and the *Ocelot catheter* (Avinger, Redwood City, CA; in use for peripheral CTOs).^49^ In the *TOC,* the *OCR* information is displayed as a waveform and a visible and audible signal warns the interventionist when the device tip approaches the blood vessel wall. A currently available *IVUS* catheter is the *Eagle Eye IVUS Catheter* (Volcano, San Diego, CA; in use peripheral and coronary CTOs).^51^ Furthermore, Webster^62^ describes a device that uses *IVUS* for clot identification in combination with a laser for crossing (see Fig. 8).

Two other imaging techniques for intravascular imaging are proposed in the patented literature using a laser^32^ and electrical impedance.^28^ In the laser approach, tissue distinction is accomplished by comparing the optical fluorescent characteristics of the excited tissue to that of known healthy and plaque tissue (see Fig. 8 for a schematic representation of this system).^32^ In the electrical impedance approach, Lafontaine *et al.*^28^ suggest using the difference in electrical impedance between the plaque and the blood vessel wall to distinguish between these two tissue types (see Fig. 8).

## Discussion

### Devices in Clinical Practice

Many of the identified devices are specifically designed for crossing total occlusions. Of the discussed devices, however, only a handful is FDA-approved and actually used in clinical practice. Even though quite promising results are achieved with the FDA-approved crossing devices, there is still improvement needed to increase the success rates in the highly calcified CTOs.

### Abandoned Crossing Devices

Even though many different devices have been developed for the treatment of heavily calcified CTOs, most of these devices never progressed beyond the investigational phase because of two main reasons. Firstly, many devices demonstrated excessively high rates of complications, particularly dissection or perforation.^55^ Secondly, the success rates of many of abandoned devices was not significantly higher than those achieved by standard crossing devices.^55^ Examples of failed CTO devices are axially rotating drill devices and several (excimer) lasers.^55^

Abandoned device designs can give important clues to the reasons for the excessive complication rate. First of all, the above-mentioned devices all exhibit an extremely limited ability to adjust to the 3D shape of the blood vessel and CTO. This makes these instruments potentially more prone to cause trauma, especially in tortuous CTOs. Secondly, lasers (and fluid jets) give limited feedback about the axial depth of the beam, making these devices more difficult to control. Thirdly, many of the axially rotating drill devices are designed with sharp edges to increase the chance of a successful crossing action, which at the same time increases the chance of blood vessel wall trauma.

Aside from directly visible damage to the blood vessel wall, other adverse events such as heat damage can also seriously impair the blood vessel wall and lead to serious complications. Lasers have been known to cause thermal damage to the surrounding tissues, warranting the slow advancement of 1 mm/s to increase absorption within the plaque.^63^ Furthermore, we assume that the proposed device relying on spark erosion^6^ and high speed rotating drills^5^,^12^,^23^,^37^,^42^ can cause heat damage to the blood vessel wall as well, which may have also have been a potential factor that led to abandonment.

It could be argued that if the benefits outweigh the risks, the device could still be used in clinical practice. Preventive measures could then be taken to minimize the risks of blood vessel wall damage. However, in most cases the devices were not only associated with an increased risk of blood vessel wall damage, but were also not very beneficial when it comes to increasing the success rate of the intervention in comparison to standard crossing devices.^55^ Success rates with lasers in coronary CTOs, for example, did not exceed 61%.^63^

### Current Crossing Devices

In recent years, promising results have been illustrated using crossing devices such as the *Frontrunner XP* and the *Crosser catheter*. Success rates as high as 90% are reported with these new crossing devices. This raises the question if there is still a need for innovative crossing devices. The answer to this question is yes.

The reported high success rates with the new crossing devices are sometimes deceptive. The highest success rates are reported in peripheral CTOs or occlusions younger than 3 months old, and are often in the hands of very experienced operators. For example, the high success rate achieved with *Frontrunner XP* and *Enabler*-*P* of 91 and 86.4%, respectively, were both achieved in peripheral vessels.^63^ Peripheral and younger CTOs are usually less calcified than coronary CTOs and thus easier to cross.^19^,^55^ This is substantiated by the fact that even though *Frontrunner XP* was originally designed for crossing coronary CTOs, more recently, the device has been increasingly used to facilitate guidewire crossing in peripheral CTOs.^9^ Furthermore, it was found that he extent of calcification increases the frequency of complications and decreases the success rate of *Frontrunner XP*.^9^

Crossing heavily calcified lesions is, therefore, still the limiting factor for success in most devices. Next to *Frontrunner XP*, the age of the CTO, calcification, and the length of the occlusion, were indicated to negatively affect the success rate in *ATD, Wildcat catheter, Safe*-*Cross,* and the *vibrational angioplasty device* (Medical Miracles, UK).^12^,^27^,^42^,^63^ Calcium is more resistant to compression and resection, and thus requires higher energy input for crossing. Therefore, to improve the success rate the energy delivery on the CTO needs to increase.

Limited directional control is also an issue in most of current crossing device designs. For example, the *vibrational angioplasty device* is considered unsuitable for smaller and tortuous vessels due to limited directional control.^63^ Additionally, *Crosser Catheter* has the tendency to move straight inside a vessel, which limits its trackability and increases the chance of entering side-branches and dissection.^63^ As coronary CTOs tend to be smaller in diameter and more tortuous than peripheral CTOs, they pose a bigger challenge. Adding the ability to actively navigate through occlusions, such as in steerable guidewires, only solves part of this problem, as visual information is still needed about the relative position of the device in relation to the CTO.

The position of the CTO (in the coronary or peripheral vasculature) is also of influence on the type of crossing device that can be applied in a safe manner. The heart is sensitive to arrhythmogenic effects caused by cavitation, vibration, and heat (for example). Therefore, the use of lasers, mechanical vibration, and cavitation for crossing coronary CTOs are relatively high risk. Since the chance of arrhythmogenic effects is minimal in crossing peripheral CTOs, the treatment options, and the use of a laser (for example), is less risky.

### Proposed Crossing Devices

Next to the previously discussed devices, many devices are proposed for crossing CTOs (amongst other occlusion types), but not (yet) seen in clinical practice or trials. Unfortunately, no information is found about the reason that these are not (yet) clinically available. Therefore, assumptions are made based on design considerations.

In general, the medical environment, including the size, shape, and biomechanical properties of the blood vessel and CTO, in which the device needs to function, should be the starting point of the design process. Lack of proper research into the medical environment can lead to device malfunction or procedural failure, which are both assumed to have occurred in several of the proposed designs.

Size restrictions of the device, based on the position of the CTO, will give important clues if certain working principles are feasible or if new issues, such as insufficient force generation, can arise. Additionally, the limitations and abilities of different manufacturing techniques should also be taken into account. Some of the described devices, such as the drive belt mechanism proposed by Thompson *et al.*,^57^ are most likely impossible to manufacture at the 1 mm diameter scale needed for the coronary application.

The biomechanical properties of the CTO and the blood vessel wall are also important factors to take into account. Since the inability to successfully cross the CTO is the most common failure mode, mainly due to buckling of the guidewire, the buckling resistance of the device should be a major factor to take into account in the design. However, high buckling resistance should not go hand in hand with an increased risk of blood vessel wall damage, as is the case in lasers and fluid jets. Since buckling and blood vessel wall trauma can both lead to procedural failure, it is important to find middle ground, which is seen in the centerline crossing devices.

Next to individual properties, the difference in biomechanical properties within the CTO itself, as well as the biomechanical difference of the CTO and blood vessel should be taken into account when designing. For example, in the crack propagation crossing method, proposed by Zakai *et al.*,^64^ it is assumed that the CTO will crack before the blood vessel wall is damaged. However, it is more likely that the pressure needed to cross a heavily calcified CTO is higher than the pressure needed to damage, unnaturally stretch, or even rupture, the blood vessel wall. Additionally, it is assumed that precise steering with the directional stiffness guidewire is difficult since the friction between the different tissue types inside the CTO will differ, resulting in varying deflections of the tip.^35^

Finally, non-design related factors that could have halted development of the proposed devices are the required high-risk and high-cost investments to develop, test, legalize, and manufacture a clinical tool. Some of the proposed devices have probably never made it to a testing phase. Which devices this involves is unclear.

### Future Crossing Device

#### Desired Crossing Path

The question remains that if a crossing device was to be developed that is technically able to cross the occlusion, what would then be the best crossing path. All the described crossing paths and associated crossing devices have their advantages and disadvantages. However, the two most important factor that needs to be taken into account when determining the best crossing path is the chance of blood vessel wall damage and buckling resistance of the crossing tool; allowing for crossing heavily calcified regions.

In order to prevent blood vessel wall trauma, it is important to be able to steer clear of the blood vessel wall. Therefore, the crossing device should be able to adapt to the direction and shape of the blood vessel. Since in the *Device* approach (*A*), the environment and user are of minimal influence on the crossing path, this crossing method is not considered feasible for integration in a future crossing device. However, preventing blood vessel wall contact and damage can be achieved in the *Environment* (*B*) and *User* (*C*) defined crossing.

The most obvious choice seems to be to give the user full control of the crossing action (*User* approach). However, the image capability, especially in 3D, of the conventional C-arcs in most clinics, is still limited for a real-time application. Therefore, it is questionable whether the interventionist would have sufficient information at hand to prevent blood vessel wall trauma.

Safe intraluminal crossing can be assured by giving the interventionist additional information with a tissue-selective imaging technique (*Sensor Enhanced Crossing*) or by adding a mechanism that ensures the device follows the path of least resistance or centerline through the CTO. Even though both approaches can assure safe crossing, another important factor that should be taken into account is the buckling resistance of the crossing device. In this respect, the *Centerline Crossing* approach is most promising, due to the increase of effective cross-sectional diameter.

Therefore, in order to ensure both safe and successful crossing of the most heavily calcified CTOs, it not only important to steer clear of the blood vessel wall, but also to prevent buckling. This can be assured by combining the user approach, in which the interventionist is able to actively navigate through the CTO and is thus able to circumnavigate heavily calcified regions, with the environment approach, where the blood vessel wall or path of least resistance is followed through the CTO. Furthermore, by adding a tissue-selective imaging technique, as in *Sensor Enhanced Crossing*, the success rate might be improved by giving the user additional information about the consistency and shape of the CTO.

#### Desired Functionality

According to the experts, if a new device for CTO crossing were to be developed it would be necessary to incorporate three functions: (1) crossing of the harder calcified regions, (2) visualization, and (3) steerability. Some crossing devices were identified that have incorporated one of these functions, such as visualization in the *Ocelot Catheter* (Avinger, Redwood City, CA).^49^ However, not a single device exists that integrates all three functionalities.

In order to achieve all three described functions in a future device it is necessary to look into the possibility of further miniaturization of the device components or to look into shared component use. In the latter approach components are used to achieve multiple functions, such as steering and crossing of heavily calcified regions. An example of a multifunctional device component is a miniature cable. Cables can be used for steering as in the steerable guidewires, visualization as in *OCT* and *IVUS*, actuators as in rotating drills and drive belt mechanisms, and for crossing as in lasers. Cables are thus very versatile, and are available in many different shapes, sizes, and materials. However, miniature cables lack buckling resistance at the required small sizes. It would, therefore, be necessary to look into additional measures to ensure sufficient buckling resistance, such as balloons or expandable structures.

It may also be possible to design a highly effective and safe crossing device without incorporating all three functionalities described by the experts. By focusing on increasing the buckling resistance and preventing blood vessel wall damage, other options, such as an inherent safe device that is able to cross the harder calcified regions and follows the blood vessel wall, may be explored.

## Conclusion

In this review, a comprehensive overview is given of current, proposed, and abandoned devices for crossing total occlusions, including CTOs. The identified crossing devices were subdivided based on their crossing paths through the occlusion and subsequently reviewed based on their ability to safely cross heavily calcified CTOs. Insight is given into reasons for abandonment of past crossing devices, needed improvement to current crossing devices, and design considerations for future crossing devices. To improve the success rate for PCI in CTOs in future it is argued that a future crossing device should be able to safely and efficiently cross the hardest CTO lesions using a combined *user* approach, in which the interventionist is able to actively navigate the crossing device, and *environment* approach, to allow for an inherent safe device with improved buckling resistance.
